# Effects of CaMKII-Mediated Phosphorylation of Ryanodine Receptor Type 2 on Islet Calcium Handling, Insulin Secretion, and Glucose Tolerance

**DOI:** 10.1371/journal.pone.0058655

**Published:** 2013-03-13

**Authors:** Sayali S. Dixit, Tiannan Wang, Eiffel John Q. Manzano, Shin Yoo, Jeongkyung Lee, David Y. Chiang, Nicole Ryan, Jonathan L. Respress, Vijay K. Yechoor, Xander H. T. Wehrens

**Affiliations:** 1 Department of Molecular Physiology and Biophysics, Baylor College of Medicine, Houston, Texas, United States of America; 2 Diabetes and Endocrinology Research Center and Department of Medicine, Division of Diabetes, Endocrinology, and Metabolism, Baylor College of Medicine, Houston, Texas, United States of America; 3 Department of Medicine, Division of Cardiology, Baylor College of Medicine, Houston, Texas, United States of America; CRCHUM-Montreal Diabetes Research Center, Canada

## Abstract

Altered insulin secretion contributes to the pathogenesis of type 2 diabetes. This alteration is correlated with altered intracellular Ca^2+^-handling in pancreatic β cells. Insulin secretion is triggered by elevation in cytoplasmic Ca^2+^ concentration ([Ca^2+^]_cyt_) of β cells. This elevation in [Ca^2+^]_cyt_ leads to activation of Ca^2+^/calmodulin-dependent protein kinase II (CAMKII), which, in turn, controls multiple aspects of insulin secretion. CaMKII is known to phosphorylate ryanodine receptor 2 (RyR2), an intracellular Ca^2+^-release channel implicated in Ca^2+^-dependent steps of insulin secretion. Our data show that RyR2 is CaMKII phosphorylated in a pancreatic β-cell line in a glucose-sensitive manner. However, it is not clear whether any change in CaMKII-mediated phosphorylation underlies abnormal RyR2 function in β cells and whether such a change contributes to alterations in insulin secretion. Therefore, knock-in mice with a mutation in RyR2 that mimics its constitutive CaMKII phosphorylation, RyR2-S2814D, were studied. This mutation led to a gain-of-function defect in RyR2 indicated by increased basal RyR2-mediated Ca^2+^ leak in islets of these mice. This chronic *in vivo* defect in RyR2 resulted in basal hyperinsulinemia. In addition, S2814D mice also developed glucose intolerance, impaired glucose-stimulated insulin secretion and lowered [Ca^2+^]_cyt_ transients, which are hallmarks of pre-diabetes. The glucose-sensitive Ca^2+^ pool in islets from S2814D mice was also reduced. These observations were supported by immunohistochemical analyses of islets in diabetic human and mouse pancreata that revealed significantly enhanced CaMKII phosphorylation of RyR2 in type 2 diabetes. Together, these studies implicate that the chronic gain-of-function defect in RyR2 due to CaMKII hyperphosphorylation is a novel mechanism that contributes to pathogenesis of type 2 diabetes.

## Introduction

Diabetes mellitus is a metabolic disease characterized by high blood glucose levels. High blood glucose levels result from either the impaired pancreatic production or secretion of, or the cellular response to insulin [1]. Of the various forms, type 2 diabetes is the most common and is characterized by inadequate insulin secretion. Insulin secretion is primarily triggered by glucose. Glucose is transported into β cells and metabolized, which increases the concentration of ATP ([ATP]). This increase in [ATP] leads to the closure of ATP-sensitive K^+^ channels and depolarization of the cellular membrane. This depolarization activates voltage-gated Ca^2+^ channels, allowing entry of extracellular Ca^2+^ into β cells, which in turn triggers a greater release of Ca^2+^ from intracellular pools [1]. The resulting elevation in cytoplasmic Ca^2+^ concentrations ([Ca^2+^]_cyt_) triggers the secretion of insulin. Thus, Ca^2+^-induced Ca^2+^ release (CICR) from intracellular pools is a critical step in the process of insulin secretion. Consequently, under type 2 diabetic conditions, defects in insulin secretion are found to be associated with alterations in intracellular Ca^2+^ handling of both rodent and human pancreatic β cells [2].

During insulin secretion, the enzyme Ca^2+^/calmodulin-dependent protein kinase II (CaMKII) is activated in response to increased [Ca^2+^]_cyt_ in β cells [3], [4]. CaMKII has also been suggested to promote Ca^2+^-dependent intracellular Ca^2+^ release [5], [6]. Easom *et al.* [7] demonstrated that the activation of CaMKII and secretion of insulin are correlated in a temporal and dose-dependent manner, while pharmacological inhibition of CaMKII diminished insulin secretion [8], [9]. In addition, CaMKII has been reported to phosphorylate several proteins, such as synapsin 1 and microtubule-associated protein-2 (MAP-2), that are involved in the trafficking and docking of insulin secretory granules during the process of insulin exocytosis [4]. Moreover, transgenic expression of CaMKIIα in mice led to impaired β cell proliferation as well as the development of insulin-dependent diabetes [10], suggesting that CaMKII may modulate insulin secretion. Therefore, it is critical to define the CaMKII-mediated regulation of pathways related to insulin secretion and identify its key downstream targets in β cells. To date, it remains unclear how Ca^2+^ handling proteins downstream of CaMKII are responsible for insulin secretion, although a member of the ryanodine receptor (RyR) family, RyR2, has been implicated as a substrate of CaMKII in pancreatic β cells [5].

Ryanodine receptors, intracellular Ca^2+^ release channels localized on the endoplasmic reticulum (ER), are the major cellular mediators of CICR in mammalian cells [11]. In β cells, various isoforms of RyRs [12], [13] are expressed, including RyR2 [14], [15]. Strong evidence suggests that RyRs are important for Ca^2+^-dependent processes in insulin secretion, although another class of intracellular Ca^2+^ release channels known as inositol triphosphate (IP3) receptors may also contribute [14]. In human β cells, acute activation of RyRs stimulates insulin secretion in a [Ca^2+^]_cyt_-dependent manner. Interestingly, acute inhibition of RyRs could also stimulate insulin secretion, albeit in a [Ca^2+^]_cyt_- and glucose-independent manner [12]. RyRs were also implicated in cAMP-dependent CICR during the process of insulin secretion, after stimulation by glucose or similar secretagogues [14]. Specifically, RyR2 was proposed as a substrate of the CD38/cADPr pathway for glucose-stimulated insulin secretion (GSIS), but this pathway was later shown to be important for β cell survival instead [16]. Furthermore, loss of FK506-binding protein 12.6 (FKBP12.6), an endogenous inhibitor of RyR2 released by cAMP activation, interfered with GSIS in two mouse model studies [17], [18], indicating an emerging role for RyR2 in the process of insulin secretion.

Although RyR2 has been implicated in insulin secretion, it is still not clear how RyR2 activity is regulated in β cells during insulin secretion. Our previous studies in cardiomyocytes revealed that RyR2 activity is regulated by CaMKII by phosphorylation at residue S2814 [11]. CaMKII-mediated phosphorylation of RyR2 was shown to rapidly activate the channel under physiological conditions [19]. On the other hand, chronic CaMKII phosphorylation of RyR2 in cardiomyocytes has been observed in disease pathology [20]. However, in pancreatic β cells, it remains to be understood whether CaMKII-mediated phosphorylation of RyR2 is involved in insulin secretion, whether any alteration in this signaling mechanism underlies abnormal RyR2 function, and whether such an alteration contributes to dysfunctional insulin secretion.

Here, we hypothesized that CaMKII phosphorylation is critical for regulating RyR2 activity in pancreatic β cells and that this pathway contributes to glucose-stimulated insulin secretion. In this present study, knock-in mice with the genetically activated CaMKII phosphorylation site (S2814) on RyR2 developed glucose intolerance and abnormal insulin secretion. These effects were associated with altered intracellular Ca^2+^ handling in pancreatic β cells. Our findings suggest that this novel mechanism of CaMKII-mediated phosphorylation of RyR2 in pancreatic β cells contributes to the development of type 2 diabetes and may aid in the development of future therapeutic targets to regulate insulin secretion under pathological conditions.

## Results

### RyR2 is the Predominant Isoform in Mouse Islets

**Figure 1:**
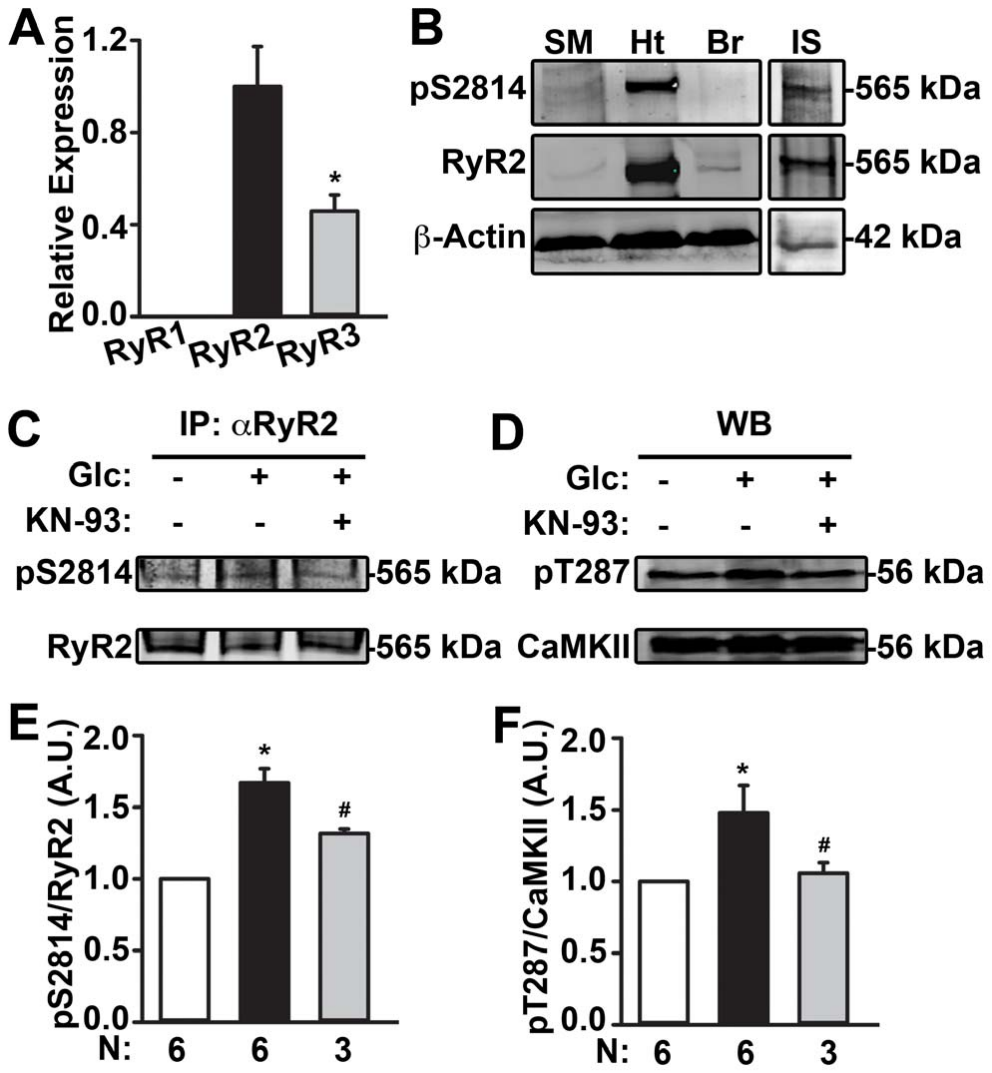
Detection of CaMKII-mediated phosphorylation on RyR2 and determination of its sensitivity to glucose in INS-1 cells. (**A**) Quantification of qRTPCR analyses showing mRNA transcript levels of RyR1, RyR2 and RyR3 in islets from WT mice (represented in arbitrary units (A.U.)). (**B**) Representative Western blots for RyR2 expression and phosphorylated RyR2-S2814 in lysates of skeletal muscles (SM), heart (Ht), and brain (Br) from WT mice and rat insulinoma INS-1 cells (IS). β-actin was used to normalize protein concentrations. Observation indicated that RyR2 was present and could be phosphorylated in IS cells, comparable to the Ht. (**C**) Representative Western blots for RyR2 and its phosphorylation at S2814 from immunoprecipitates obtained from IS lysates using anti-RyR2 antibody. The lysates were prepared from INS-1 cells incubated with 2.8 mM (−) glucose or stimulated with 25 mM (+) glucose with and without pre-treatment with 10 µM KN-93. (**D**) Representative Western blots for CaMKII and its autophosphorylation at T287 from IS lysates generated in the above experiments. (**E**) Quantification revealed increased S2814 phosphorylation (pS2814) normalized to total RyR2 level upon glucose (25 mM) stimulation. Pre-treatment with 10 µM KN-93 blunted this increase. (**F**) Quantification also revealed increased autophosphorylation of CaMKII at T287 (pT287) normalized to total CaMKII level upon stimulation with 25 mM glucose but not in the presence of KN-93. Data (N = 3–6 experiments) are represented as average ± SEM. *P<0.05, vs. 2.8 mM glucose; ^#^ *P*<0.05 vs. 25 mM glucose.

To determine the predominant RyR isoform found in pancreatic islets, mRNA levels of RyR1, RyR2, and RyR3 were determined using quantitative RT-PCR (qRT-PCR). Primer specificity for each isoform was confirmed in various tissues that were enriched in only one or two RyR isoforms. RyR1 mRNA was detected only in skeletal muscle, whereas RyR2 was detected in both cardiac and brain tissue of wild type (WT) mice. RyR3 was also detected in the brain (**Figure S1A**). In murine pancreatic islets both RyR2 and RyR3 mRNA but not RyR1 mRNA were detected (**Figure 1A** **)**. However, the normalized expression level of RyR2 was significantly higher than that of RyR3 (1.00±0.09 vs. 0.46±0.04 A.U.; *P*<0.05). Similar to previous studies [14], [15], our qRT-PCR results demonstrated that RyR2 is indeed the most predominant RyR isoform in pancreatic islets. Moreover, these data are in agreement with our Western blot data obtained from rat insulinoma-derived glucose-responsive β cells (INS-1 cell line). Not only was RyR2 present but we were also able to detect RyR2 phosphorylation at residue S2814 by CaMKII in both cardiac and INS-1 cells (**Figure 1B**). These data not only confirm the presence of RyR2, but also demonstrate that CaMKII can phosphorylate RyR2 in β cells.

### Increase in CaMKII Phosphorylation of RyR2 Upon Glucose Stimulation in INS-1 Cells

After glucose stimulation, RyR2 was immunoprecipitated from INS-1 cell lysates and Western blotting was conducted on the immunoprecipitates using phosphoepitope-specific antibodies. These experiments revealed an increase in CaMKII phosphorylation of S2814 on RyR2 in INS-1 cells stimulated with 25 mM glucose compared to those incubated with 2.8 mM glucose (1.67±0.10 vs. 1.00 A.U.; *P*<0.05) (**Figure 1C and E** **)**. Global CaMKII activity, assessed by Western blotting for autophosphorylation of CaMKII at T287 [19] in the glucose-stimulated INS-1 cell lysates, was also increased (1.48±0.19 vs. 1.00 A.U.; *P*<0.05) (**Figure 1D and F** **)**. Moreover, pretreatment with the CaMKII inhibitor KN-93 significantly reduced the glucose-induced increase in RyR2 phosphorylation at S2814 by 48.4±2.2% and CaMKII autophosphorylation at T287 by 88.1±10.2% (**Figure 1C–F**). Furthermore, immunoblotting experiments in lysates of islets from WT mice revealed a 20% increase in autophosphorylation levels of CaMKII at T287 normalized to total CaMKII level upon stimulation with 25 mM glucose (**Figure S2A**). These data indicate a positive correlation between glucose stimulation, CaMKII activation, and CaMKII-mediated phosphorylation of RyR2 in pancreatic β cells.

### Calcium Leak in Mice with Chronic CaMKII Phosphorylation of RyR2

Since our data demonstrated a link between CaMKII-mediated phosphorylation of RyR2 and glucose-stimulated insulin secretion, we next investigated whether alterations in CaMKII-mediated phosphorylation of RyR2 cause abnormal Ca^2+^ channel function in β cells as well as defects in insulin secretion. To study the specific effects of increased RyR2-mediated Ca^2+^ release, we studied RyR2-S2814D knock-in mice (S2814D) [21] in which the CaMKII phosphorylation site on RyR2 is constitutively activated.

**Figure 2:**
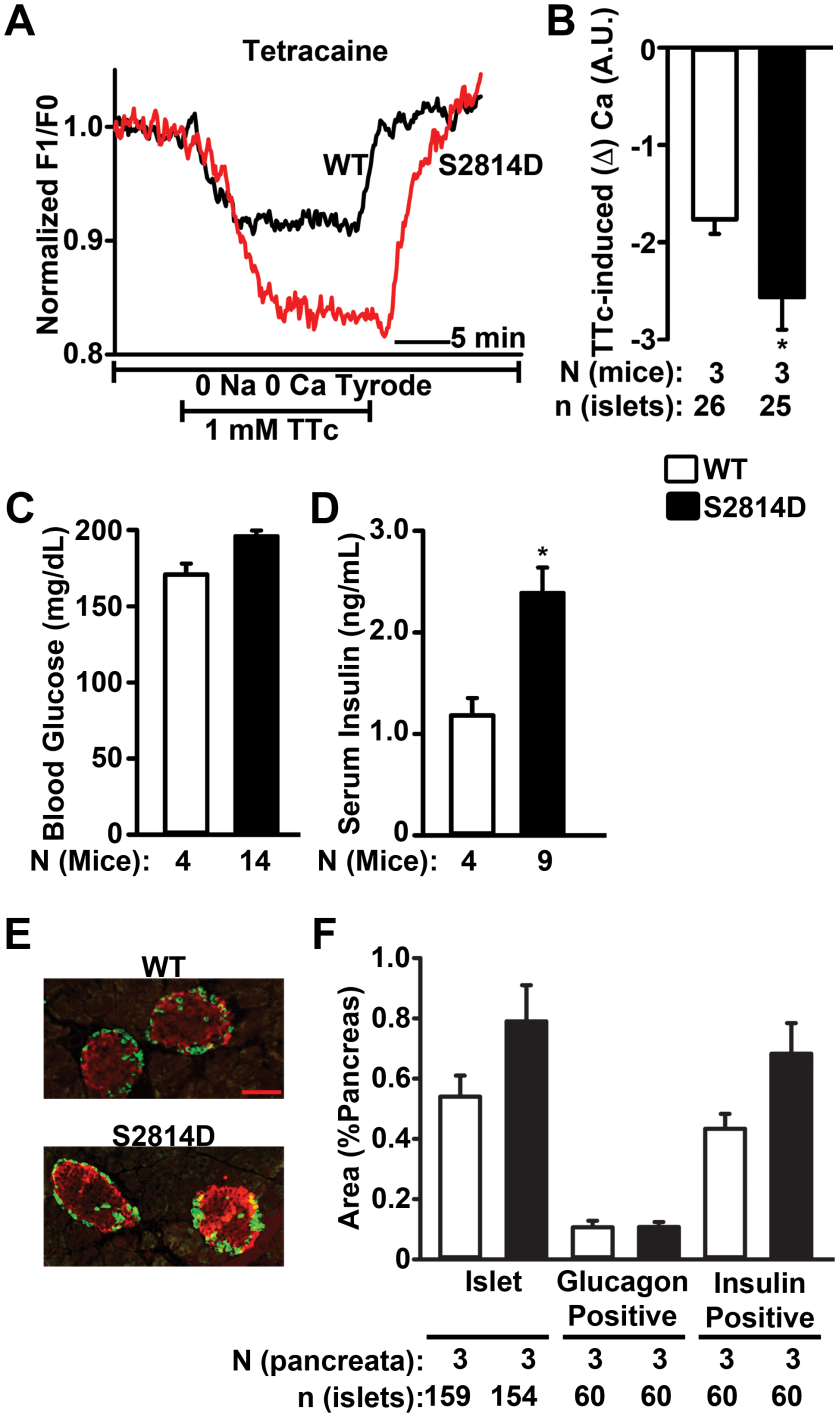
RyR2-mediated Ca^2+^ leak and basal hyperinsulinemia in S2814D mice. (**A**) Representative cytoplasmic Ca^2+^ level ([Ca^2+^]_cyt_) tracings from islets following perfusion with 1 mM tetracaine (TTc). (**B**) Quantification of decrease in [Ca^2+^]_cyt_ (represented in arbitary units (A.U.)) (greater Ca^2+^ leak) in S2814D islets compared to WT islets in response to 1 mM TTc. n>25 islets from 3 mice per group. (**C**) Average blood glucose levels in WT (N = 4) and S2814D (N = 14) mice after 6 h fasting. (**D**) Average serum insulin levels in WT (N = 4) and S2814D (N = 9) mice after 6 h fasting. Note higher baseline insulin levels in S2814D mice. (**E**) Representative immunohistochemical images of frozen pancreas sections from WT and S2814D mice. Islets were stained with glucagon (*green*) and insulin (*red*). Scale bar, 100 µm. (**F**) Quantification of percentage area of pancreatic tissue occupied by total islets or glucagon- or insulin-positive islet fractions in WT and S2814D mice. Number of mice (pancreata) and total number of islets studied indicated below the bars. Data are represented as average ± SEM. *P<0.05 vs. WT.

Pancreatic islets isolated from WT and S2814D mice were loaded with a Ca^2+^ sensitive dye and SR Ca^2+^ leak was measured using the tetracaine (TTc) protocol described by Shannon *et al.* [22]. TTc has been shown to rapidly and reversibly block RyR2, promoting Ca^2+^ uptake into the SR/ER from the cytoplasm. This TTc-dependent shift of Ca^2+^ is measured as the decrease in [Ca^2+^]_cyt_ in the absence of extracellular Ca^2+^, and is proportional to total SR/ER Ca^2+^ leak. Following its application, TTc induced a larger decrease in [Ca^2+^]_cyt_ (greater Ca^2+^ leak) in islets from S2814D mice (2.56±0.3 arbitrary units (A.U.)) compared to WT islets (1.76±0.2 A.U.; *P*<0.05) (**Figure 2A and B**), suggesting a major role for S2814 phosphorylation on RyR2 in β cells. Thus, mutation S2814D on RyR2 results in a chronic gain-of-function defect in RyR2 in pancreatic β cells.

To study the effect of this gain-of-function defect in RyR2 on insulin secretion, we measured fasting blood glucose and insulin levels in WT and S2814D mice. Following 6 hours of fasting, WT and S2814D mice displayed similar blood glucose levels (178.0±10.2 mg/dL vs 168.0±9.0 mg/dL, respectively; *P* = NS) (**Figure 2C**). Both the groups of mice had similar serum glucagon levels (**Figure S3A**). However, fasting insulin levels were significantly higher in S2814D mice (2.23±0.3 ng/mL) compared to WT mice (1.18±0.2 ng/mL; P<0.01) (**Figure 2D**), suggesting that chronic activation of RyR2 at S2814 increases the basal secretion of insulin *in vivo*.

To determine whether mutation S2814D in RyR2 has any effect on pancreatic structure and thereby causes basal hyperinsulinemia, we performed histological sectioning of pancreata from WT and S2814D mice. Total islet area (**Figure S3B**) and the percent pancreatic area occupied by islets were similar in both S2814D (0.79±0.1%) and WT (0.54±0.1%; *P* = NS; **Figure 2E and F**) mice as revealed by immunostaining. In addition, the glucagon- (0.11±0.02% S2814D vs. 0.11±0.03% WT; *P* = NS) and insulin-positive areas (0.43±0.05% vs. 0.68±0.10%; *P* = NS) were also similar in size in both S2814D and WT pancreata (**Figure 2F**). Moreover, biochemical analyses revealed similar islet insulin contents in islets isolated from both groups (**Figure S3C**). Furthermore, mRNA levels of key genes that play a role in insulin secretion as well as Ca^2+^ regulation, including that of RyR2, were also similar in islets from WT and S2814D mice (**Figure S1B–C**). Thus, mutation S2814D in RyR2 does not alter pancreatic structure or gene expression. Therefore, the observed phenotype of basal hyperinsulinemia in S2814D mice is primarily due to the gain-of-function defect in RyR2.

### Defective Glucose Metabolism in S2814D Mice

**Figure 3:**
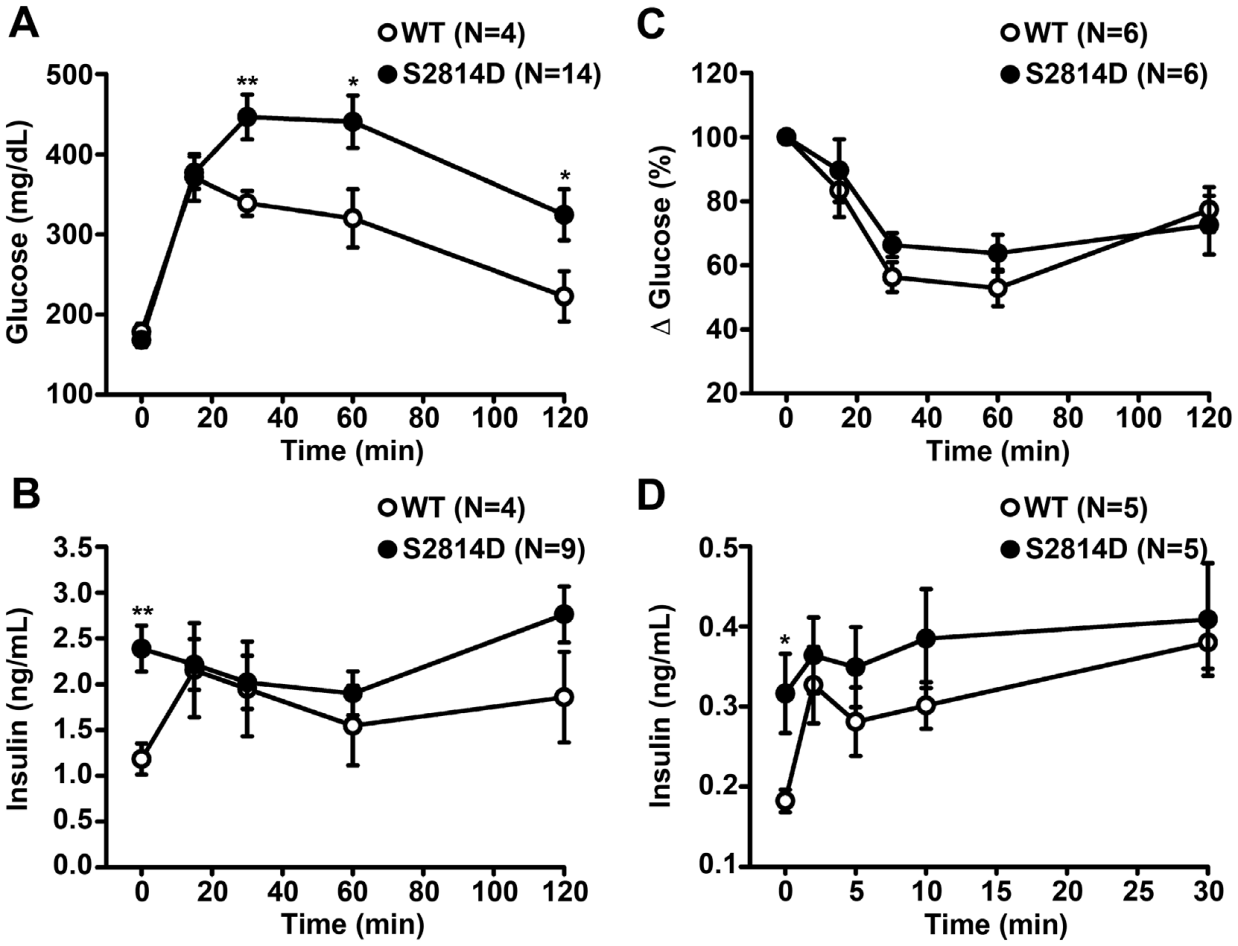
Glucose intolerance and impaired glucose-induced insulin secretion in S2814D mice. (**A, B**) Glucose tolerance tests were conducted by injecting glucose (2 g/kg body weight) in WT and S2814D mice after a period of fasting for 6 hours. Quantifaction revealed glucose intolerance in S2814D mice as evidenced by higher blood glucose levels (**A**) and the lack of an insulin response (**B**). (**C**) Insulin tolerance tests were conducted by injecting insulin (0.75 U/kg body weight) after 6 h of fasting. (**D**) Acute phase insulin secretion in response to glucose (3 g/kg body weight) after 16 hours of fasting revealed a blunted response in S2814D mice. Data are represented as average ± SEM. N = 4−14 mice per group. **P<0.01, *P<0.05 vs. WT.

Next, we determined whether basal hyperinsulinemia could affect the ability of S2814D mice to clear excess blood glucose. Therefore, glucose tolerance tests (GTT) were conducted in WT and S2814D mice. S2814D mice exhibited significantly higher blood glucose levels than WT mice, starting at 30 min after glucose injection (2 g glucose/kg), (**Figure 3A**) and continued to display significantly high blood glucose levels (324.4±31.9 mg/dL) compared to WT mice (222.6±31.4 mg/dL; P<0.05) up to 2 hours post injection. Following 30 min after the glucose challenge, WT mice produced a 60±20% increase in serum insulin levels (**Figure 3B**), whereas insulin levels in S2814D mice did not significantly deviate from the already higher basal levels.

Because serum insulin levels and glucose tolerance might be altered due to peripheral insulin resistance, we then measured insulin sensitivity in S2814D mice using the insulin tolerance test (ITT). The blood-glucose concentrations dropped to similar extents in WT and S2814D mice, when normalized to their respective baseline values, at 30 min post insulin injection (0.75 U insulin/kg) (**Figure 3C**). After 2 hours, blood glucose levels had recuperated in both WT and S2814D mice, suggesting that S2814D mice retained insulin sensitivity and that abnormal glucose metabolism in S2814D mice was not due to peripheral insulin resistance.

### Defective Acute Glucose-stimulated Insulin Secretion in S2814D Mice

The insulin secretory dysfunction observed in S2814D mice during the 2 hour-long GTT mainly represents defects in the secretion of newly formed insulin granules, which is the sustained phase of insulin secretion [1]. However, secretion of pre-formed insulin granules, the acute phase of insulin secretion, is typically initiated within 2 min after glucose stimulation, and lasts only for a few minutes [23]. To characterize the acute phase of insulin secretion in S2814D mice, an important pre-indicator of β cell dysfunction [1], acute GSIS was assayed after a longer fasting period (16 hours) and following a higher glucose dose (3 g/kg body weight) than the previous GTT. The basal insulin levels were still significantly higher in S2814D mice even after 16 hours fasting. Upon administration of the glucose dose, WT mice showed a 79.4% increase in insulin concentrations after 2 min (**Figure 3D**). In contrast, S2814D mice exhibited a blunted (15.0%) increase (*P*<0.05) above their higher basal insulin levels. Thus, S2814D mice exhibited impairments in both the acute and the sustained phases of GSIS.

### Impaired *in vitro* Glucose-stimulated Insulin Secretion in Islets from S2814D Mice

**Figure 4:**
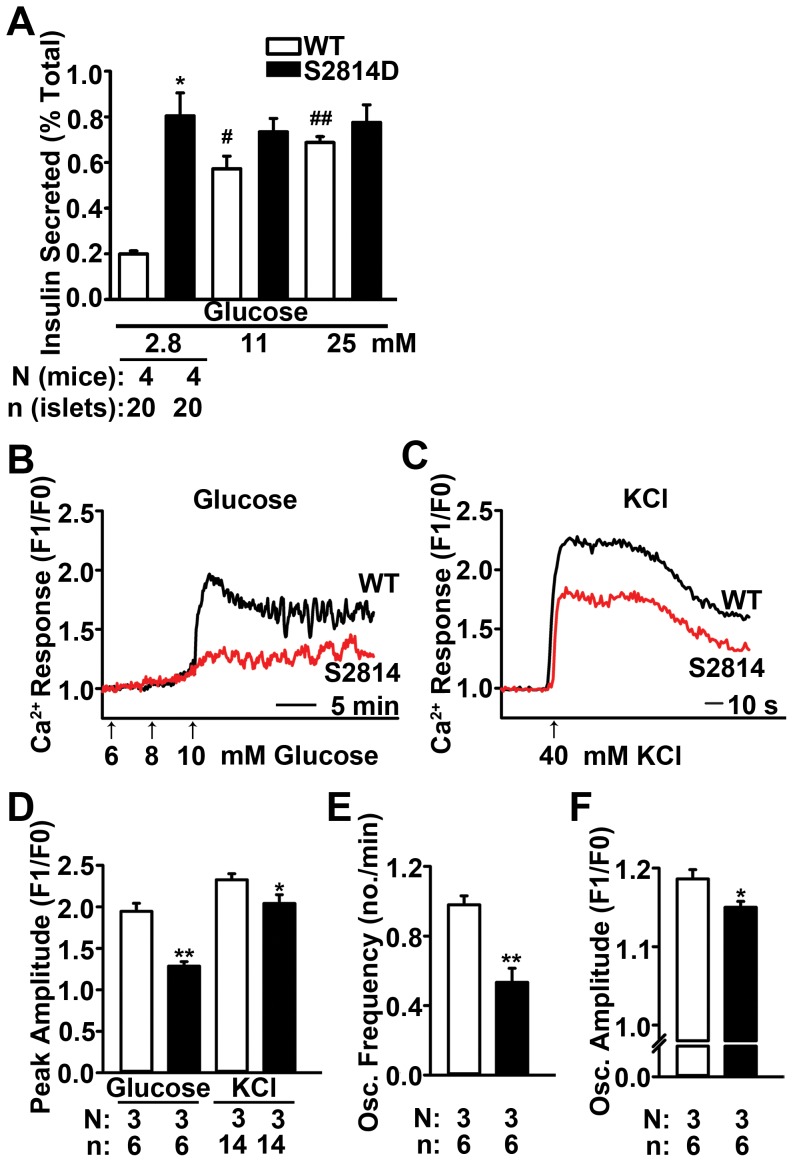
Defective glucose-stimulated insulin secretion and Ca^2+^ transient in S2814D mice. (**A**) Insulin secretion from 4 sets of 5 islets each (each set from a different mouse, n = 20 islets from 4 mice per group) during 30-minute sequential exposures to glucose (2.8, 11 and 25 mM). Quantification revealed higher basal insulin secretion in islets from S2814D mice, which did not increase in response to glucose stimulation, compared to WT islets. (**B**) Representative tracings of [Ca^2+^]_cyt_ in WT and S2814D islets following glucose stimulation at varying concentrations (6, 8, and 10 mM). (**C**) Representative [Ca^2+^]_cyt_ tracings following stimulation with 40 mM KCl. (**D**) Quantification revealed blunted [Ca^2+^]_cyt_ transient amplitude in response to 10 mM glucose or 40 mM KCl in islets from S2814D mice compared to WT. In addition, frequency of [Ca^2+^]_cyt_ oscillations (**E**) and amplitude of [Ca^2+^]_cyt_ oscillations following initial peak (**F**) after 10 mM glucose stimulation were also reduced in S2814D islets. n = 6−14 islets from 3 mice per group. Data are represented as average ± SEM. *P<0.05, **P<0.01 vs. WT; ^#^ *P*<0.05, ^##^ *P*<0.01 vs. 2.8 mM glucose.

To further characterize the insulin secretory defect, an *in vitro* GSIS assay was conducted in islets isolated from S2814D mice. In the presence of 2.8 mM glucose, islets from S2814D mice secreted significantly higher insulin amounts (0.8±0.1% of total insulin content) as compared to islets from WT mice (0.20±0.01%; *P*<0.05) (**Figure 4A**). Stimulation with 11 and 25 mM glucose, respectively, led to 0.93±0.09 and 0.95±0.03 fold increases over the basal insulin secretion in islets from S2814D mice (*P* = NS for both vs. S2814D at 2.8 mM). These increases were significantly less than those seen in islets from WT mice (2.86±0.19 and 3.47±0.24, *P*<0.05 and *P*<0.01 vs. WT at 2.8 mM) (*P*<0.01 for both vs. respective S2814D values). Thus, in agreement with our *in vivo* metabolic data, islets from S2814D mice also exhibit defects in insulin secretion.

### Impaired Glucose-stimulated Ca^2+^ Transient in Islets from S2814D Mice

As mentioned previously, insulin secretion is driven by changes in [Ca^2+^]_cyt_. Upon glucose stimulation, [Ca^2+^]_cyt_ is elevated as a result of both Ca^2+^ entry from outside the cell across the plasma membrane and Ca^2+^ release from the ER [1]. Therefore, islets from WT and S2814D mice were studied to assess whether mutation S2814D in RyR2 also alters the response in [Ca^2+^]_cyt_ to glucose stimulation [24],. Upon stimulation with 10 mM glucose, [Ca^2+^]_cyt_ in islets from WT and S2814D mice rose significantly to a peak and exhibited oscillations thereafter (**Figure 4B**). However, the amplitude of the initial peak of [Ca^2+^]_cyt_ was smaller in islets from S2814D mice (1.29±0.1) compared to those from WT mice (1.95±0.1; *P*<0.01) (**Figure 4D**). Moreover, the frequency (and also amplitude) of the subsequent [Ca^2+^]_cyt_ oscillations was reduced in islets from S2814D mice (0.53±0.1 per min) compared to islets from WT mice (0.98±0.1 per min, *P*<0.01) (**Figure 4E and F**). These data indicate that the glucose-stimulated Ca^2+^ transient is impaired in islets from S2814D mice, as seen in type 2 diabetic patients [**25**].

Furthermore, stimulation with KCl, an insulin secretagogue that acts downstream of glucose [26], elicited very rapid and short [Ca^2+^]_cyt_ responses in islets from both S2814D and WT mice, with a peak occurring at 10 s after addition of KCl and a total response lasting for <2 min (**Figure 4C**). However, the peak amplitude of KCl-stimulated [Ca^2+^]_cyt_ was also significantly lower in islets from S2814D mice (2.01±0.1) compared to those from WT mice (2.33±0.1; *P*<0.05) (**Figure 4D**). Thus, islets from S2814D mice exhibit a defect downstream of the KCl-mediated depolarization of the plasma membrane during insulin secretion.

### Reduced Glucose-sensitive Intracellular Ca^2+^ Pool in Islets from S2814D Mice

**Figure 5:**
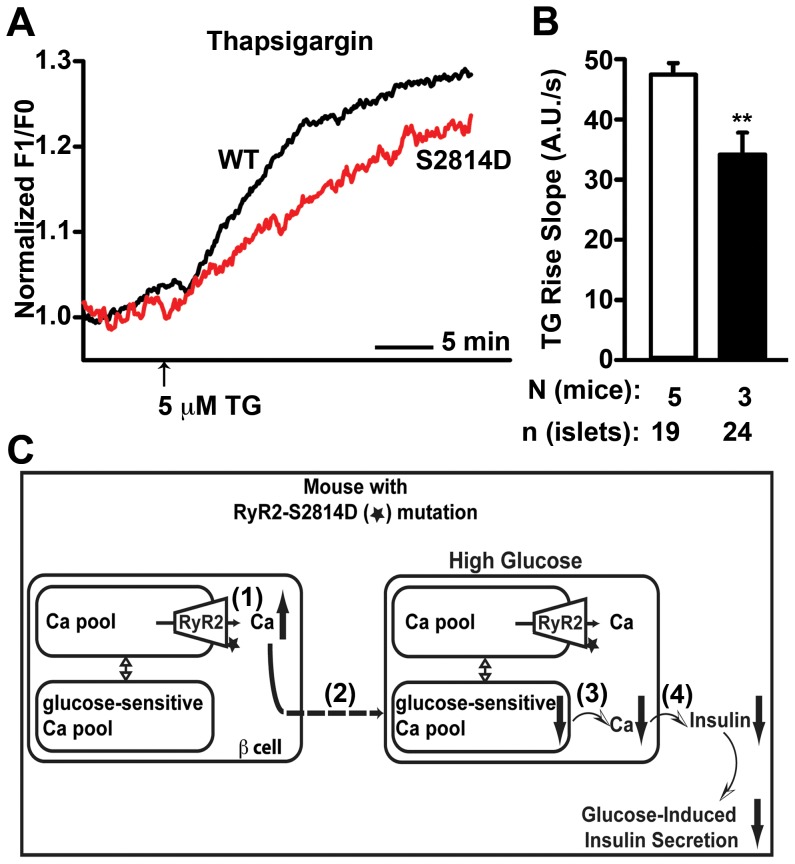
Reduced glucose-sensitive Ca^2+^ pools and the putative mechanism underlying glucose-metabolism defects in S2814D mice. (**A**) Representative [Ca^2+^]_cyt_ tracings in islets isolated from WT and S2814D mice following stimulation with 5 µM thapsigargin (TG). (**B**) Quantification of [Ca^2+^]_cyt_ transient rise per sec in response to 5 µM TG in islets from WT and S2814D mice. Data are represented as average ± SEM. n = 19−24 islets from 3–5 mice per group. **P<0.01 versus WT. (**C**) Mutation S2814D results in reduced glucose-sensitive Ca^2+^ pools in islets from S2814D mice. (1) Due to S2814D mutation, RyR2 leaks Ca^2+^ from intracellular Ca^2+^ stores in pancreatic β cells. (2) Chronic Ca^2+^ leak, in turn, leads to (dotted arrow) a decrease (downward arrow) in the glucose-sensitive Ca^2+^ pool in S2814D mice. (3) This decrease in the Ca^2+^ pool is reflected by a decrease in glucose-stimulated intracellular Ca^2+^ transients, which (4) blunts glucose-stimulated insulin secretion in S2814D mice.

Given that expression levels of Ca^2+^ channels on the plasma membrane are unaltered and RyR2 is located on the ER membrane, the defective glucose-stimulated [Ca^2+^]_cyt_ elevation in islets from S2814D mice likely arises due to defective Ca^2+^ release from the ER due to RyR2-mediated Ca^2+^ leak. Therefore, we also investigated ER Ca^2+^ properties in islets from S2814D mice. Although strong evidence suggests that the intracellular Ca^2+^ pools are in equilibrium with each other [27], at present, there is no consensus about the role of RyR2 in the release of the glucose-sensitive Ca^2+^ pool of β cells [5], [12], [13], [28]. Previous studies have demonstrated that the glucose-sensitive Ca^2+^ pool is sensitive to thapsigargin (TG) [29], [30], a blocker of sarco/endoplasmic reticulum Ca^2+^ ATPase (SERCA), which prevents Ca^2+^ sequestration into intracellular Ca^2+^ pools. To assess the effects of the gain-of-function defect in RyR2 on the size of glucose-sensitive Ca^2+^ pool - by definition the most relevant pool for glucose-stimulated insulin secretion – we measured the [Ca^2+^]_cyt_ response to TG in islets from WT and S2814D mice [31]. Both WT and S2814D islets displayed an increase in [Ca^2+^]_cyt_ in response to TG (**Figure 5A**). However, in response to TG, cytosolic Ca^2+^ increase in islets from S2814D mice was significantly slower than in islets from WT mice as reflected by a reduction in slope (47.46±1.9 vs. 34.20±3.6 A.U./s, *P*<0.01) (**Figure 5B**). These data strongly suggest a reduction in glucose-sensitive Ca^2+^ pools in β cells of S2814D mice, similar to reported findings from other type 2 diabetic models [30]. Overall, our findings suggest that the RyR2-mediated Ca^2+^ leak in islets from S2814D mice lowers the amount of Ca^2+^ storage in β cells, resulting in defective glucose-stimulated Ca^2+^ handling and insulin release from β cells (**Figure 5C**).

### Increased CaMKII Phosphorylation of RyR2 in Human and Mouse Type 2 Diabetes

**Figure 6:**
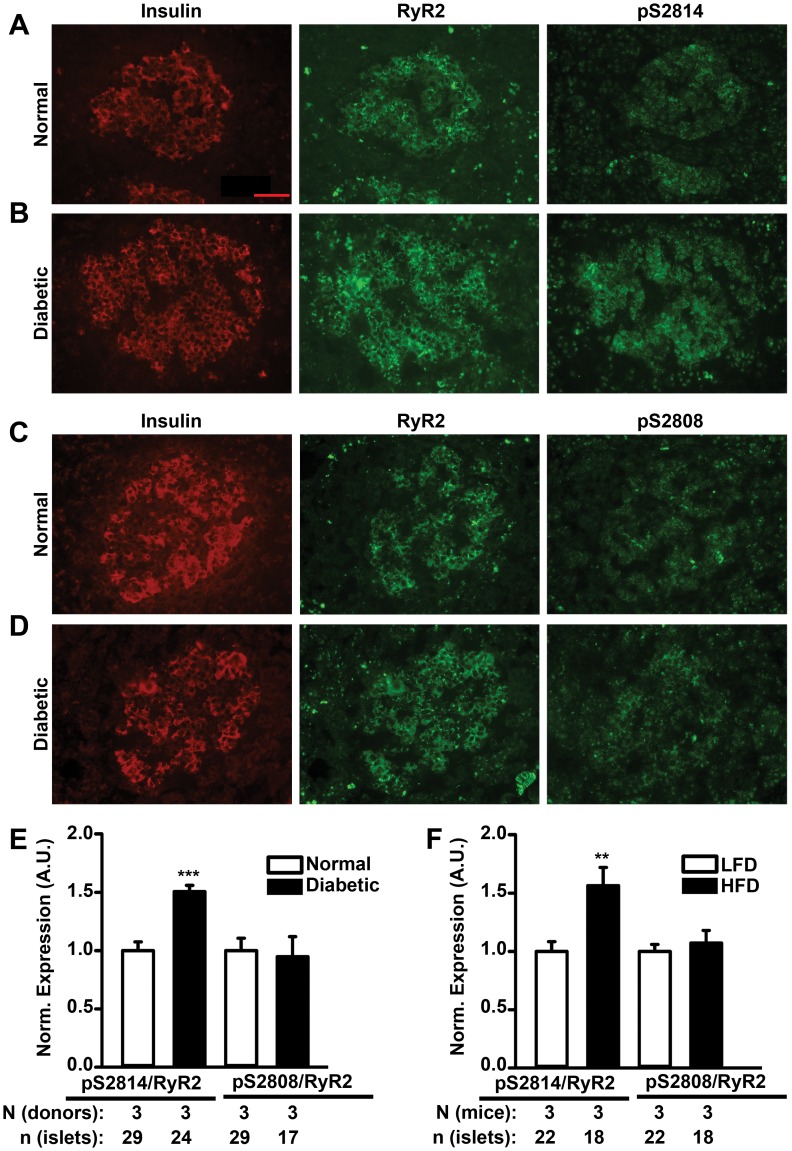
Increased CaMKII phosphorylation of RyR2 in type 2 diabetes. (**A, B**) Immunolabeling of sequential frozen human pancreatic sections from normal donors and type 2 diabetic donors revealed similar total RyR2 levels in β cells, but increased S2814 phosphorylation (pS2814) of RyR2 in diabetic donors. (**C, D**) Immunolabeling showing similar levels of S2808 phosphorylation (pS2808) of RyR2 in human β cells from normal and diabetic donors. Scale bars, 50 µm. (**E**) Bar graph showing quantification of S2814 and S2808 phosphorylation, normalized to total RyR2 signal, respectively, in human islets. Quantification revealed increased S2814 phosphorylation in human islets isolated from diabetic donors. n = 17−29 islets from 3 donors per group. ***P<0.001 versus healthy donors. (**F**) Quantifications of RyR2 phosphorylation at S2814 and S2808 normalized to total RyR2 signal, respectively, also demonstrated increased levels of phosphorylated S2814 (pS2814) in high fat diet-fed HFD (type 2 diabetic) mice compared to low fat diet-fed (LFD) mice. Data are represented as average ± SEM. n = 17−29 islets from 3 donors per group, n = 18−22 islets from 3 mice per group. ***P<0.001 vs. normal donor, **P<0.01 vs. LFD.

The aforementioned observations raise the question of whether the mechanism of chronic CaMKII phosphorylation of RyR2 is pathophysiologically relevant. Therefore, RyR2 phosphorylation levels were measured in pancreatic β cells of normal and type 2 diabetic human donors using immunohistochemical analyses. In addition to CaMKII-mediated phosphorylation, levels of protein kinase A (PKA)-mediated phosphorylation of RyR2 at residue S2808, another phosphorylation site that is known to rapidly modulate RyR2 activity [32], were also measured. Although total RyR2 expression levels were unaltered, phosphorylation levels at S2814 on RyR2 were significantly increased in islets from type 2 diabetic donors compared to healthy donors (1.50±0.1 vs. 1.00±0.1; *P*<0.001) (**Figure 6A–B and E**). In contrast, there were no significant changes in S2808 phosphorylation in islets from type 2 diabetic donors compared to those from normal donors (0.95±0.2 vs. 1.00±0.1 *P* = NS) **(** **Figure 6C–E**).

Since it was difficult to control for the severity of type 2 diabetes in human donors, RyR2 immunolabeling studies were repeated in a mouse model of obesity-associated type 2 diabetes induced by high fat diet (HFD) [33] (**Figure S4A**). In our studies, HFD-fed mice were sacrificed when they started developing insulin resistance (**Figure S4C**), with moderately high (not severe) blood glucose levels (**Figure S4B**). At this stage, possible confounding effects from systemic glucotoxicity were minimal [34]. β cells of these HFD-fed mice exhibited significantly higher S2814 phosphorylation of RyR2 (1.56±0.2 vs. 1.00±0.1; *P*<0.01) compared to β cells of low-fat diet (LFD)-fed mice (**Figure 6F**). No increase was observed in S2808 phosphorylation on RyR2 (1.07±0.1 vs. 1.00±0.1; *P* = NS) in β cells of the HFD-fed mice.

For ensuring validity of the imunohistochemical analyses, specificities of the antibodies - anti-RyR2 (Millipore, Billerica, MA) and phospho-epitope specific anti-pS2814-RyR2 and anti- pS2808-RyR2 antibodies (custom-made) - were checked by biochemical and immunohistochemical analyses in RyR2-positive and negative tissues and by checking specificities of the antigen peptides *in silico* (details in the Materials and Methods section). Specifically, the anti-pS2814 antibody was generated using the peptide C-SQTSQV-(pS)-VD [19], [56], unlike commonly used non-specific phospho-Ser antibodies that are generated only against the specific amino acid. The antigen peptide corresponds to CaMKII phosphorylation site on RyR2 at Ser 2814 and is unique to RyR2 (NCBI-Protein-BLAST search analyses). The specificity of the antibody was established in previous studies by Western blotting [21], [56]. So far, it has not been possible to crosscheck specificity of the antibody in immunohistochemical studies, implicating a potential limitation of the analysis due to possible cross-reactivity of the antibody towards other proteins.

The immunohistochemical data were complemented by immunoblotting experiments in lysates of islets from LFD and HFD mice. The analyses revealed a 25% increase in autophosphorylation levels of CaMKII at T287 normalized to total CaMKII level in the obesity-induced type 2 diabetic condition in HFD mice (**Figure S2B**). These data suggest that CaMKII and its downstream target RyR2 are activated in type 2 diabetic conditions.

## Discussion

Insulin secretion from pancreatic islets depends on a tightly modulated process of intracellular Ca^2+^ handling. However, the presence and role of the intracellular Ca^2+^ release channel RyR2 in insulin secretion has not been well established [14]. Previous studies have demonstrated that acute activation of RyRs increases [Ca^2+^]_cyt_ and stimulates basal insulin secretion in human β cells [12]. Conversely, acute inhibition of RyRs could also stimulate insulin secretion in a [Ca^2+^]_cyt_- and glucose-independent manner. Based on these observations, it was postulated that RyR2 did not play a major role in GSIS, although some studies suggested a role for RyR2 in GSIS in an cAMP-dependent manner [2].

Our findings provide clear evidence that RyR2 is the most predominant RyR isoform present in mouse islets. We show that not only is RyR2 present in β cells, but that it is also regulated by CaMKII-mediated phosphorylation in a glucose-sensitive manner. Thus, our data support the notion that phosphorylation of RyR2 may be involved in the process of GSIS.

Unlike previous studies, where RyR2 was either acutely activated or inhibited *in vitro*, our study utilized an *in vivo* model of RyR2 activation, namely the constitutively activated RyR2-S2814D knock-in mouse [21]. This model has been shown to exhibit a chronic, *in vivo* gain-of-function defect in RyR2 that results in a significant RyR2-mediated Ca^2+^ leak and basal hyperinsulinemia. Basal hyperinsulinemia, in itself, can be an early predictor of type 2 diabetes [35]. Moreover, S2814D knock-in mice developed glucose intolerance, a hallmark of pre-diabetic conditions [1]. Furthermore, S2814D mice displayed loss of both acute and sustained phases of GSIS as seen in type 2 diabetic patients [36], implying a severe abnormality in β cell function. As a result, S2814D mice presented with alterations in glucose homeostasis, before elevation of fasting blood glucose levels, ruling out the possibility of secondary effects due to systemic glucotoxicity. The apparent differences between our studies and previous studies are most likely due to the different ways in which RyR2 activity is modulated in respective experimental models.

Moreover, previous studies [8], [9] have shown that acute CaMKII inhibition diminishes insulin secretion in cultured cells or islets. Based on these studies, it can be expected that activation of CaMKII and/or its downstream targets increases insulin secretion. In agreement, our mice with mutation S2814D in RyR2 exhibited basal hyperinsulinemia *in vivo* (**Figure 2D**) and *in vitro* (**Figure 4A**). Furthermore, these mice displayed a significant reduction in the intracellular Ca^2+^ pool (**Figure 5A–B**) as well as impaired glucose-stimulated insulin secretion. Thus, the single amino acid change in RyR2 at the CaMKII phosphorylation site can mimic the expected effect of CaMKII activation on insulin secretion. These data support our conclusion that RyR2 is a key target through which CaMKII regulates insulin secretion in β cells.

As shown in **Figure 4**, the gain-of-function defect in RyR2 in islets from S2814D mice significantly decreased the amplitude of glucose-stimulated [Ca^2+^]_cyt_ transients, as well as the frequency and amplitude of glucose-stimulated [Ca^2+^]_cyt_ oscillations. Similar observations have been made in type 2 diabetic patients, where alterations in glucose-stimulated Ca^2+^ oscillations [25] were closely related to abnormalities in the pattern of insulin secretion [36]. These studies suggest that the chronic defect in RyR2 due to CaMKII hyperphosphorylation might alter the pattern of insulin secretion and contribute to the development of pre-diabetic conditions in S2814D mice.

In our model, defects in GSIS can be explained by intracellular Ca^2+^ store properties in islets from S2814D mice. Strong evidence suggests that the intracellular Ca^2+^ pools are in equilibrium with each other [27]. However, it is not clear whether RyR2 is a part of the glucose-sensitive Ca^2+^ pool in β cells [5], [12], [13], [28]. Therefore, we demonstrated reductions in the glucose-sensitive Ca^2+^ pool without the use of any agent that sensitizes or inhibits RyR2. Irrespective of RyR2’s intracellular localization, it is important to note that a chronic gain-of-function defect in RyR2 negatively influences the glucose-sensitive Ca^2+^ pool in β cells. As a result, this reduction impairs glucose-stimulated Ca^2+^ transients and insulin secretion (as shown in **Figure 5C**). This reduction in the Ca^2+^ pool of β cells is similar to the reduction observed in other type 2 diabetic models [30].

In addition to internal release of Ca^2+^ in β cells, entry of extracellular Ca^2+^ through L-type Ca^2+^ channels (LTCC) [1], extrusion of cytosolic Ca^2+^ out of Na^+^/Ca^2+^ exchanger (NCX) [37], and reuptake of cytosolic Ca^2+^ into the sarco/endoplasmic reticulum via SERCA [29] are critical in the regulation of insulin secretion. In islets from S2814D mice, expression levels of these key Ca^2+^ channels were not altered (**Figure S1C**). These data support our conclusion that defects in insulin secretion in S2814D mice are probably due to chronic phosphorylation of RyR2 by CaMKII. However, in the case of chronic diabetes, compensatory mechanisms such as enhanced SERCA expression, upregulation of LTCC activity [38], and activation of store-operated Ca^2+^ (SOC) channels [39] may change the dynamics of Ca^2+^ homeostasis in islets as the intracellular Ca^2+^ pools starts to empty. Ultimately, the fate of β cells, under diabetic conditions, will depend on the balance between Ca^2+^ release and uptake.

In addition to insulin secretion, RyR2 has also been implicated in β cell survival [40], [41]. Johnson *et al.* [40] suggested that short-term activation of RyR2 might enhance β cell survival. Moreover, chronic activation of RyR2 might increase [Ca^2+^]_cyt_, triggering the activation of apoptotic pathways [42]. In addition, RyR2-mediated ER Ca^2+^ leak leads to reduction of intracellular Ca^2+^ pools. Such a reduction in intracellular Ca^2+^ pools could result in heightened ER stress [41], which might also trigger apoptotic pathways [43], [44]. Moreover, apoptosis arising from abnormalities in ER Ca^2+^ homeostasis (or ER stress) has been associated with diabetes [45], [46]. In our study, however, S2814D mice showed intact pancreata and islets (**Figures 2E–F and S** **3A–C**), indicating no alteration in β cell survival. We studied S2814D mice when they were pre-diabetic. At this stage RyR2 activation and RyR2-mediated Ca^2+^ leak might not have been large enough to influence the β cell survival pathways. However, in chronic type 2 diabetes chronic activation of RyR2 by CaMKII phosphorylation might contribute to the pathogenesis in two ways - by altering insulin secretion and by altering β cell survival.

The pathogenesis of type 2 diabetes is not fully understood. The main defects that lead to onset of type 2 diabetes in humans are β cell dysfunction and insulin resistance [47]. Patients with the same insulin sensitivity show either normal or impaired glucose tolerance with or without hyperglycemia. In these patients, the severity of abnormalities in the glucose metabolism is closely dependent on the degree of β cell dysfunction [47], [48]. Thus, β cell dysfunction is a critical factor in the pathogenesis of type 2 diabetes. This observation was supported by an independent study in Pima Indians where progress from normal to impaired glucose tolerance to fully-developed type 2 diabetes was correlated with β cell dysfunction [49].

To understand the molecular mechanisms underlying pathogenesis of human type 2 diabetes, various animal models have been effectively used. In many models type 2 diabetes stems from obesity-induced insulin resistance [50]. As a result, the molecular mechanisms that underlie β cell dysfunction at the onset of human type 2 diabetes are not well studied. Our study in the S2814D mouse model elucidates the gain-of-function defect in RyR2 due to CaMKII hyperphosphorylation as a novel mechanism that alters insulin secretion due to β cell dysfunction, thereby contributing to the development of type 2 diabetes. This conclusion is supported by the significant increase in CaMKII-mediated phosphorylation of RyR2 in β cells from human type 2 diabetic donors and from our obesity-associated type 2 diabetic (HFD) mouse model. The shift from normal to hyperphosphorylation of RyR2 in the human and mouse β cells can originate from constitutive Ca^2+^-dependent activation of CaMKII [51]. Once initiated, the loop of Ca^2+^-dependent activation of CaMKII, subsequent RyR2 phosphorylation and RyR2-associated Ca^2+^ leak might self amplify and progress in severity.

The implications of our findings for the broader scientific and medical community are that normalizing β cell dysfunction by targeting CaMKII-phosphorylation of RyR2 might normalize [Ca^2+^]_cyt_ in pancreatic β cells. This strategy might prove valuable in regulating insulin secretion and hence, slowing development of type 2 diabetes by itself and/or in combination with existing therapies.

## Materials and Methods

### Animals

RyR2-S2814D knock-in mice were generated as previously described [21]. All studies were conducted in 4 to 10-month old mice. To induce obesity-related type 2 diabetes, 5-month old C57Bl6 mice were fed on a high fat diet (HFD) (energy kcal %: protein 18.3, fat 45, carbohydrates 35.5; Testdiet, Richmond, IN) for an 8-week period and compared with age-matched C57Bl6 mice fed on a low fat diet (LFD) (energy kcal %: protein 18.3, fat 10.2, carbohydrates 71.5; Testdiet, Richmond, IN). All animal studies were performed according to protocols approved by the Institutional Animal Care and Use Committee of Baylor College of Medicine, and followed guidelines provided by the Guide for the Care and Use of Laboratory Animals published by the US National Institute of Health (No. 85–23, revised 1996).

### Immunohistochemistry

Frozen pancreatic sections from normal human adults and diabetic donors were purchased from Biochain (Hayward, CA). Mouse pancreatic sections were frozen, fixed, permeabilized and blocked. For both human and mouse sections, two sequential pancreatic sections of 5–10 µm thickeness were placed on glass slides. The sections were incubated with required combinations of rabbit polyclonal anti-glucagon (MP Biomedicals, Solon, OH), guinea pig polyclonal anti-insulin (Dako Inc., Carpinteria, CA), rabbit polyclonal anti-RyR2 (Millipore, Billerica, MA), phospho-epitope specific rabbit polyclonal anti-pS2814-RyR2 or anti-pS2808-RyR2 antibodies (custom-made), and species-specific fluorescent secondary antibodies (Invitrogen Corp., Carlsbad, CA). The anti-RyR2 antibody is a purified rabbit polyclonal antibody generated using a 15–20 amino acid synthetic peptide from the variant trans-membrane region of human RyR2 that is unique to RyR2 (AB9080, Certificate of Analysis, Millipore, Billerica, MA and NCBI-Protein-BLAST search analyses (data not shown)). Specificity of the anti-RyR2 antibody was confirmed in RyR2-expressing and non-expressing tissues by Millipore using immunohistochemical analyses and Western blotting and also by us using Western blotting (data not shown). The anti-RyR2 antibody has been widely used to specifically label RyR2 in previously published immunohistochemical analyses [52]–[54]. Furthermore, specificity of the antibody was also indicated in the Human Protein Atlas [55], where the antibody was shown to specifically stain islets but not exocrine glandular cells in immunohistochemical analyses on human pancreatic sections. The anti-pS2814 and anti-pS2808 antibodies are rabbit polyclonal antibodies generated using the peptides C-SQTSQV-(pS)-VD and C-RTRRI-(pS)-QTSQV respectively [19], [56]. The antigen peptides of the antibodies correspond to CaMKII phosphorylation site at Ser 2814 and PKA phosphorylation site at Ser 2808 on RyR2. Both the antigen peptides are unique to RyR2. The specificities of anti-pS2814 and anti-pS2808 antibodies were established in previous studies [21], [56]. After incubation with antibodies, the sections were mounted and counterstained with DAPI for nuclei.

For both human and mouse sections, immunolabeling was visualized using an epifluorescence microscope (Axiovision A1, Zeiss, Munich, Germany). Acquired images were analyzed by either software by Axiovision or image J (ImageJ Data Acquisition Software, National Institutes of Health, Bethesda, MD) [57]. For measuring the RyR2 phosphorylation level at S2814 in islets, the pancreatic sections were stained with anti-insulin antibody and double-labeled with either anti-RyR2 or anti-pS2814-RyR2 antibodies. Immunostained pancreatic sections were quantified for phospho and total RyR2 signals using Axiovision software. This software was used to delineate insulin immunoreactive islets from non-immunoreactive regions of the pancreatic sections. The non-immunoreactive regions were considered as the background. The densitometric mean value of this background was subtracted from the densitometric mean value of the insulin immunoreactive islet regions, obtained from immunostaining with either anti-RyR2 or anti-pS2814 antibodies. Normalized phospho-RyR2 values from islet regions were compared to normalized total-RyR2 values from corresponding islet regions in the sequential pancreatic section on the same slide to determine the ratio of phospho-S2814-RyR2 to total RyR2 in each islet. Similarly, the ratio of phospho-S2808-RyR2 to total RyR2 was estimated in islets of pancreatic sections using the anti-RyR2 and phospho-epitope specific anti-pS2808-RyR2 antibody.

### Cell Culture

INS-1 cells were maintained in culture as described [58].

### Glucose Stimulation Experiments

Cultured INS-1 cells or mouse islets (5.5 mM glucose) were pre-incubated with 2.8 mM glucose in RPMI for 2 hours and then stimulated with 25 mM glucose for 10 min. A subset of INS-1 cells was pre-treated with 10 µM KN-93 for a period of 30 min before glucose stimulation. Stimulated INS-1 cells were washed, harvested in PBS and spun down. Both INS-1 cells and islets were lysed by sonication in 1× RIPA buffer solution that contained protease inhibitors, phosphatase inhibitors, and 1% CHAPS. Lysates were then used for immunoprecipitation and immunoblotting assays.

### Immunoprecipitation and Western Blotting

Immunoprecipitation and Western blotting of islet lysates were conducted as previously described [21], [59].

### Quantitative Real Time Polymerase Chain Reaction (qRTPCR)

Quantitative RTPCR was conducted as described before [59]. Briefly, total RNA extracted from islets was reverse transcribed using Superscript II reverse transcriptase and oligo(dT) primer (Invitrogen, Carlsbad, CA). Real-time PCR and fluorescence detection was performed in duplicates in 96-well plates using SYBR Green and a Mastercycler ep realplex (Eppendorf, Hamburg, Germany). Expression levels were compared using the relative Ct method - the amount of target is normalized to the amount of endogenous control ( β actin) and to the control sample.

### Glucagon Measurements

After a 6-hour fasting period, the blood serum samples were collected from mice. The glucagon levels were measured in the samples using an enzyme immunoassay (R&D Systems, Minneapolis, MN).

### Glucose and Insulin Tolerance Tests

After a 6-hour fasting period, the mice were administered intraperitoneal (IP) injections of either 2 g glucose/kg body weight or 0.75 U insulin/kg body weight. Blood glucose levels were measured at 0, 15, 30, 60 and 120 min and serum insulin levels (only for GTT) were measured using ELISA kits (Mercodia, Uppsala, Sweden). For the acute phase insulin secretion assay, a higher dose of glucose (3 g/kg body weight) was administered via IP injection following a 16-hour period of fasting. Blood glucose and serum insulin levels were measured at 0, 2, 5, 10 and 30 min post injection. For LFD and HFD mice, insulin tolerance was measured under non-fasting conditions via IP injection of 1 U insulin/kg body weight.

### Islet Isolation

Islets were isolated as described by Li *et* al. [60].

### Insulin Secretion Assay

Following isolation, islets were cultured overnight in RPMI medium containing 5 mM glucose. Four of five sets islets were plated and washed with glucose-free Kreb-ringer buffer (KRB) and pre-incubated with KRB containing 2.8 mM glucose for 30 min. Next, islets were incubated with 11 and 25 mM glucose for 30 min, sequentially. At the end of each experiment, islet insulin was extracted using 0.2 M acid and ethanol. Concentrations of total and secreted insulin were measured using insulin ELISA kits (Mercodia, Uppsala, Sweden) and normalized to islet DNA content.

### Calcium Imaging

Islets were washed with glucose-free Tyrode solution and loaded with Ca^2+^ sensitive indicator 5 µM fluo-4-AM (Molecular Probes, Carlsbad, CA) for 20 min at 33°C. Intracellular [Ca^2+^]_cyt_ were recorded using a laser confocal microscope (LSM510 Zeiss, Munich, Germany) in frame-scan imaging mode. Islets were subjected to increasing glucose concentrations: 0 mM glucose for 2 min, 6 mM for 5 min, 8 mM for 5 min, and 10 mM for 30 min. Alternatively, islets were perfused with 40 mM KCl, 1 mM tetracaine, or 5 µM thapsigargin. Tetracaine experiments were conducted in 0 Na, 0 Ca^2+^ tyrode solution. Ca^2+^ signals were recorded then analyzed using Image J.

### Statistics

All data are represented as average ± SEM. Statistical significance of differences between experimental groups was determined using Student’s t-test. A value of *P*<0.05 was considered statistically significant.

## Supporting Information

Figure S1 **Gene expression analyses in WT and S2814D mice. (A)** Primer specificities of the following genes were tested in lysates prepared from skeletal muscles, heart, and brain of WT mice: RyR1, RyR2 and RyR3 viz. type 1, 2 and 3 ryanodine receptors, respectively. **(B–C)** Quantification of qRTPCR analyses showing relative mRNA expression of key insulin secretory genes in islets from WT and S2814D mice. Glut2, glucose transporter 2; Sur1, ATP-binding cassette, sub-family C; Kir6.2, potassium channel 6.2; Gcg, glucagon; Gck, glucokinase; Pdx1, pancreatic and duodenal homeobox 1; Sst, somatostatin; Neurod1, neuronal differentiation 1; Ppy, pancreatic polypeptide; IP3R, inositol 1,4,5-trisphosphate receptor; Cav1.2, voltage-gated calcium channel; NCX, sodium calcium exchanger; SERCA, SR/ER Ca^2+^ ATPase; and RyR2, type 2 ryanodine receptor. Data are presented as mean±SEM. *P* = NS.
(TIF)

Figure S2 **Increase in CaMKII autophosphorylation upon glucose stimulation and in diabetic condition. (A)** Western blot analyses for CaMKII and its autophosphorylation at T287 from WT mouse islet lysates. The Western blotting revealed 20% increase in the autophosphorylation of CaMKII at T287 normalized to total CaMKII level upon stimulation with 25 mM glucose. For this experiment, islets from 10 mice were pooled and equally divided in 2 groups for the specified experimental conditions (N = 1 experiment). **(B)** Western blot analyses for CaMKII and its autophosphorylation at T287 in lysates of islets pooled from 8 low-fat diet fed (LFD) and 8 high-fat diet fed (HFD) mice. Immunoblotting showed 25% increase in the auto phosphorylation of CaMKII at T287 normalized to total CaMKII level in the obesity-induced type 2 diabetic condition in HFD mice (N = 1 experiment).
(TIF)

Figure S3 **Normal glucagon levels and intact islets in S2814D mice. (A)** Average serum glucagon levels in WT (N = 5) and S2814D (N = 5) mice after 6 h fasting. The glucagon levels were measured using an enzyme immunoassay (R&D Systems, Minneapolis, MN). **(B)** Quantification of absolute islet areas in WT and S2814D mice. Number of mice (pancreata) and total number of islets studied indicated below the bars. **(C)** Insulin content of 5 similar-sized islets per WT and S2814D mice were measured using ELISA (Mercodia, Uppsala, Sweden) and normalized to respective DNA contents of islets. Data are presented as mean±SEM. *P* = NS.
(TIF)

Figure S4 **Higher body weights and fasting blood glucose levels but no significant insulin intolerance in high-fat diet fed mice.** **(A–B)** 20-week old C57Bl6 male mice were fed HFD (45% fat) for 8 weeks. After 8 weeks, body weights **(A)** and overnight fasting blood glucose levels **(B)** of HFD mice were compared with age-matched controls fed on low-fat diet fed (LFD). HFD mice showed significantly higher body weights and fasting blood glucose levels as compared to LFD mice. **(C)** Non-fasting insulin tolerance test was conducted by injecting insulin (1 U/kg body weight) in LFD and HFD mice. Data are presented as mean±SEM. **P<0.01, * *P*<0.05, WT vs. S2814D.
(TIF)
