# Supplementary figures and images for: Effects of CaMKII-Mediated Phosphorylation of Ryanodine Receptor Type 2 on Islet Calcium Handling, Insulin Secretion, and Glucose Tolerance
Source: PLoS One. 2013 Mar 13;8(3):e58655. doi: 10.1371/journal.pone.0058655 (PMC3596297; doi:10.1371/journal.pone.0058655)

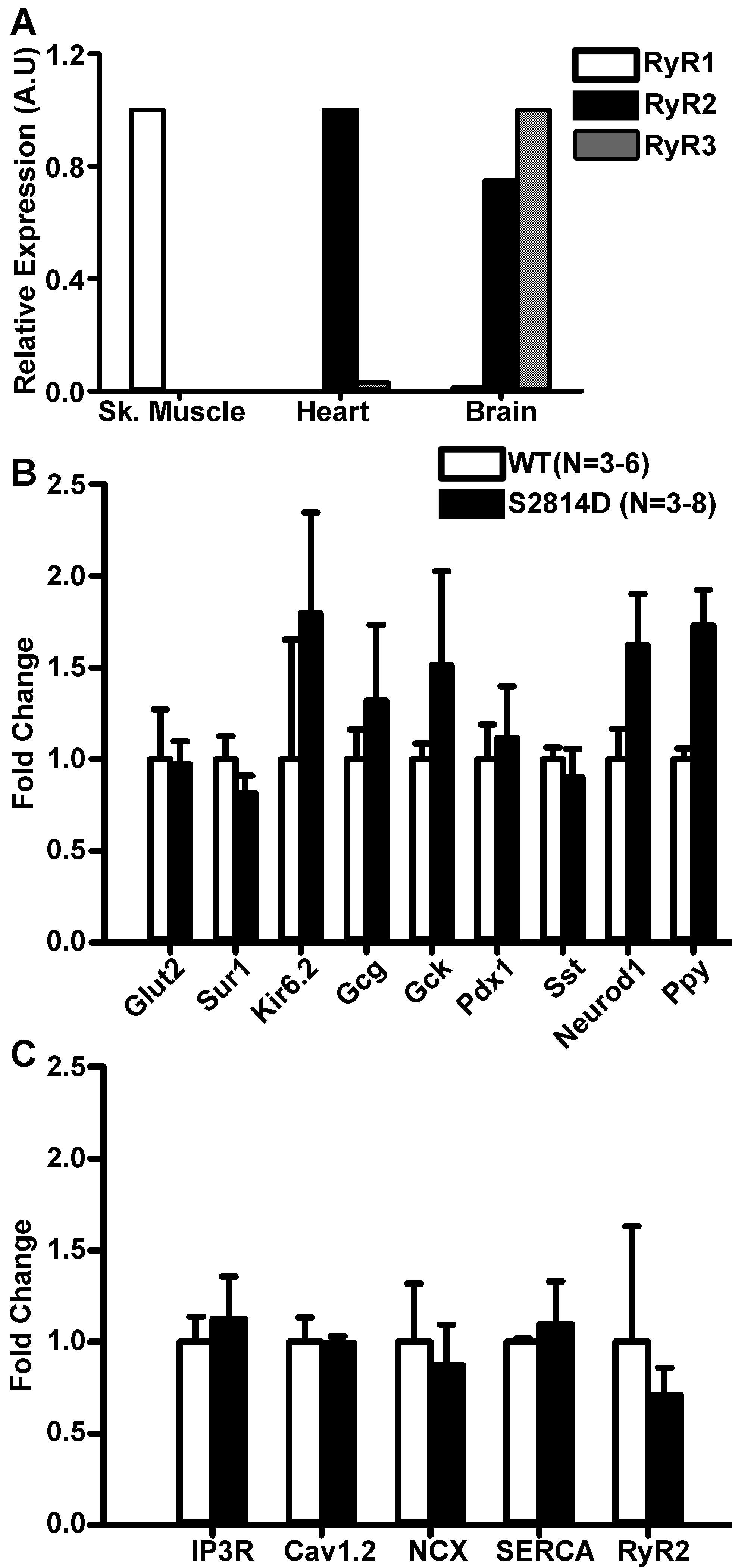

Supplement: Figure S1 — Gene expression analyses in WT and S2814D mice. (A) Primer specificities of the following genes were tested in lysates prepared from skeletal muscles, heart, and brain of WT mice: RyR1, RyR2 and RyR3 viz. type 1, 2 and 3 ryanodine receptors, respectively. (B–C) Quantification of qRTPCR analyses showing relative mRNA expression of key insulin secretory genes in islets from WT and S2814D mice. Glut2, glucose transporter 2; Sur1, ATP-binding cassette, sub-family C; Kir6.2, potassium channel 6.2; Gcg, glucagon; Gck, glucokinase; Pdx1, pancreatic and duodenal homeobox 1; Sst, somatostatin; Neurod1, neuronal differentiation 1; Ppy, pancreatic polypeptide; IP3R, inositol 1,4,5-trisphosphate receptor; Cav1.2, voltage-gated calcium channel; NCX, sodium calcium exchanger; SERCA, SR/ER Ca2+ ATPase; and RyR2, type 2 ryanodine receptor. Data are presented as mean±SEM. P = NS. (TIF) [file pone.0058655.s001.tif]

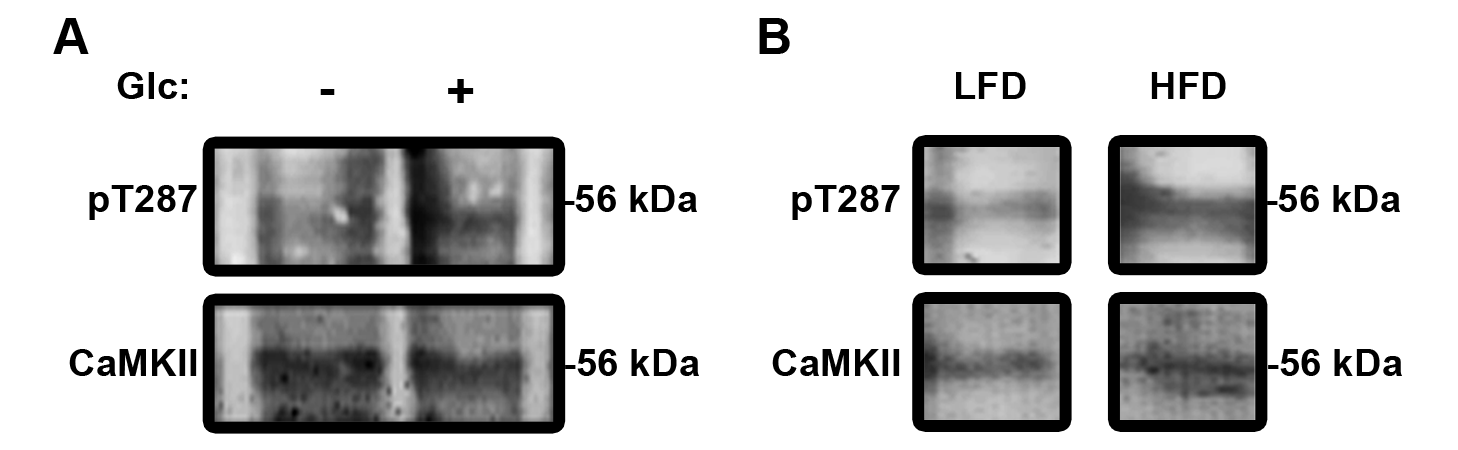

Supplement: Figure S2 — Increase in CaMKII autophosphorylation upon glucose stimulation and in diabetic condition. (A) Western blot analyses for CaMKII and its autophosphorylation at T287 from WT mouse islet lysates. The Western blotting revealed 20% increase in the autophosphorylation of CaMKII at T287 normalized to total CaMKII level upon stimulation with 25 mM glucose. For this experiment, islets from 10 mice were pooled and equally divided in 2 groups for the specified experimental conditions (N = 1 experiment). (B) Western blot analyses for CaMKII and its autophosphorylation at T287 in lysates of islets pooled from 8 low-fat diet fed (LFD) and 8 high-fat diet fed (HFD) mice. Immunoblotting showed 25% increase in the auto phosphorylation of CaMKII at T287 normalized to total CaMKII level in the obesity-induced type 2 diabetic condition in HFD mice (N = 1 experiment). (TIF) [file pone.0058655.s002.tif]

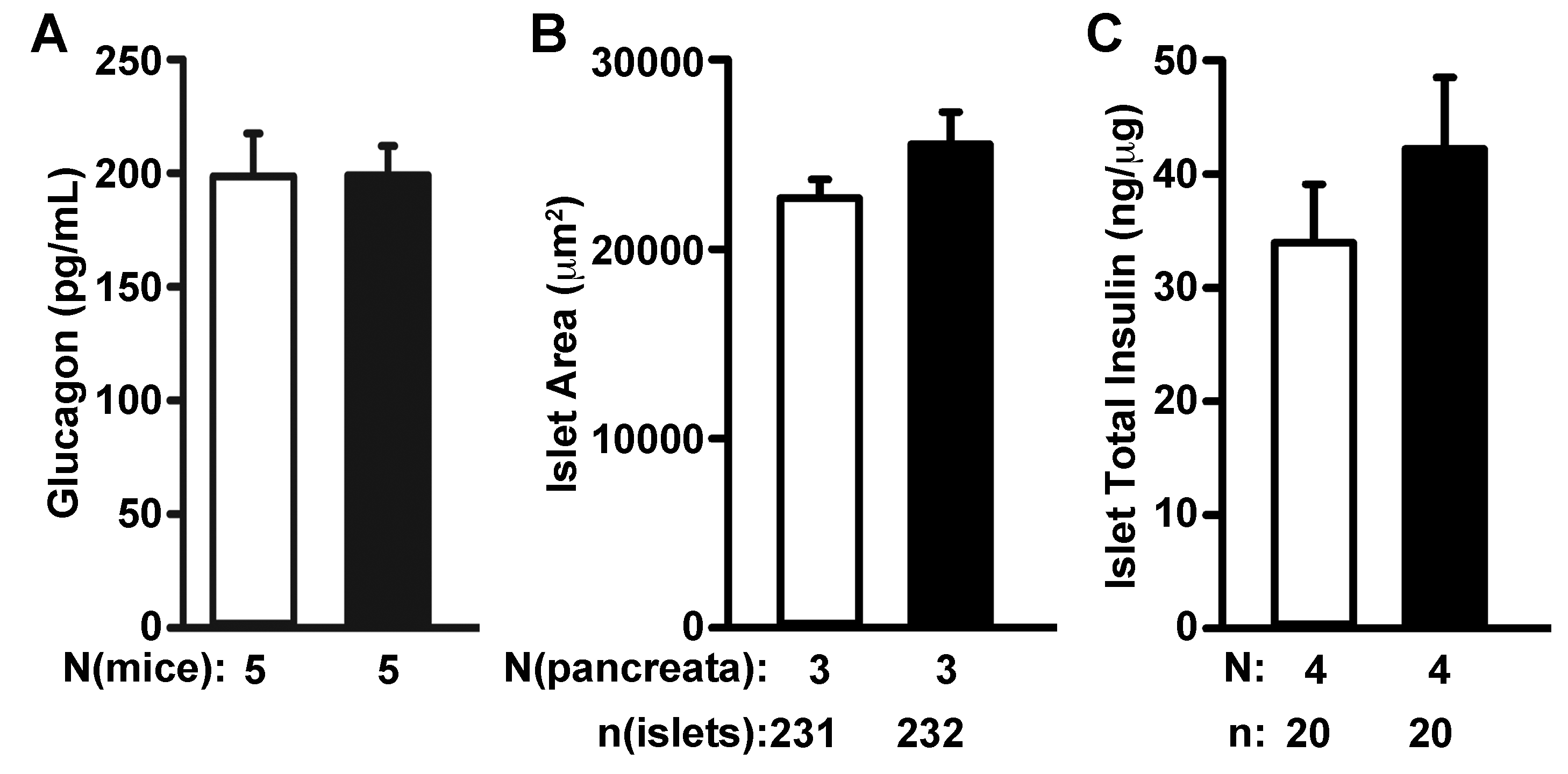

Supplement: Figure S3 — Normal glucagon levels and intact islets in S2814D mice. (A) Average serum glucagon levels in WT (N = 5) and S2814D (N = 5) mice after 6 h fasting. The glucagon levels were measured using an enzyme immunoassay (R&D Systems, Minneapolis, MN). (B) Quantification of absolute islet areas in WT and S2814D mice. Number of mice (pancreata) and total number of islets studied indicated below the bars. (C) Insulin content of 5 similar-sized islets per WT and S2814D mice were measured using ELISA (Mercodia, Uppsala, Sweden) and normalized to respective DNA contents of islets. Data are presented as mean±SEM. P = NS. (TIF) [file pone.0058655.s003.tif]

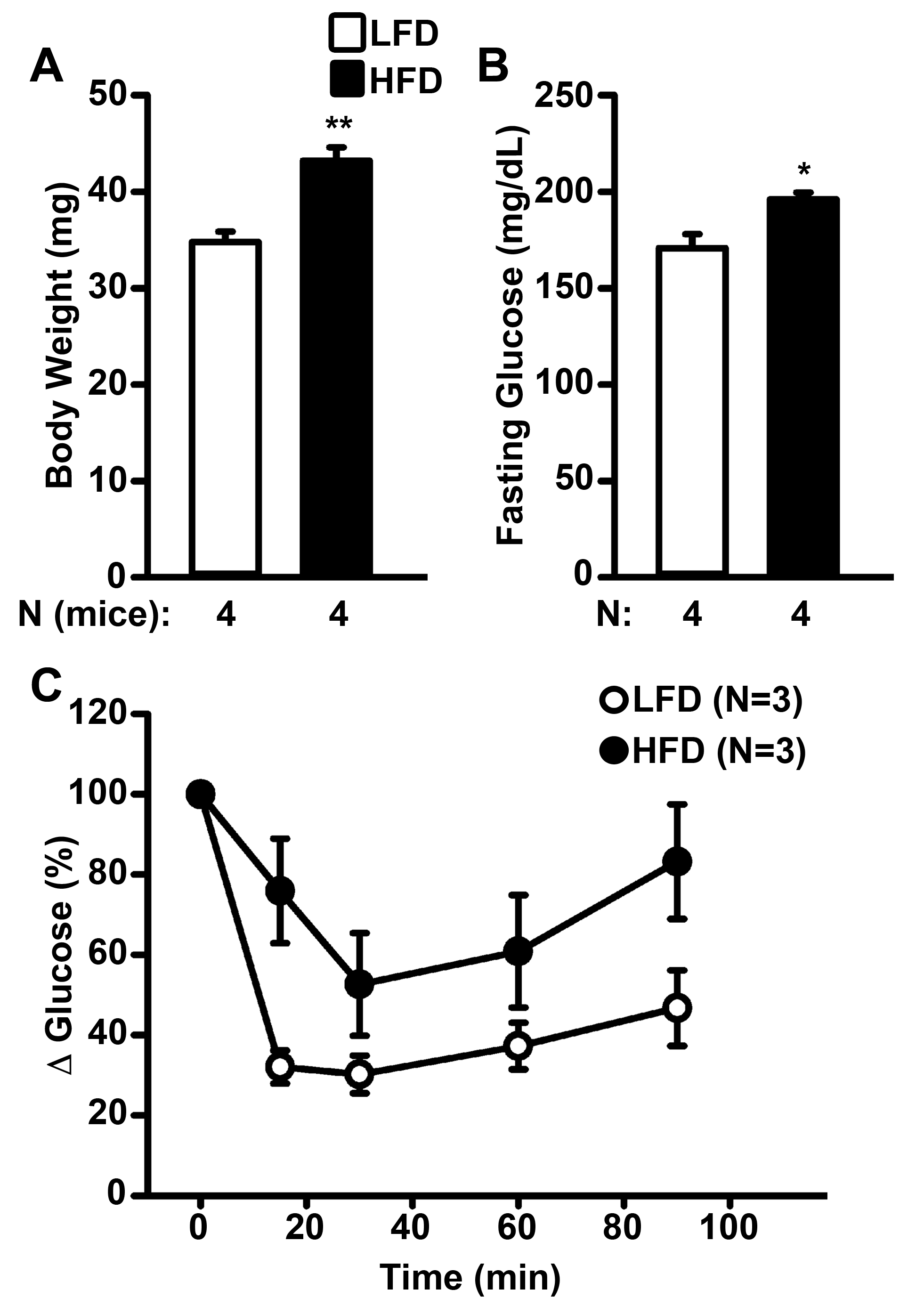

Supplement: Figure S4 — Higher body weights and fasting blood glucose levels but no significant insulin intolerance in high-fat diet fed mice. (A–B) 20-week old C57Bl6 male mice were fed HFD (45% fat) for 8 weeks. After 8 weeks, body weights (A) and overnight fasting blood glucose levels (B) of HFD mice were compared with age-matched controls fed on low-fat diet fed (LFD). HFD mice showed significantly higher body weights and fasting blood glucose levels as compared to LFD mice. (C) Non-fasting insulin tolerance test was conducted by injecting insulin (1 U/kg body weight) in LFD and HFD mice. Data are presented as mean±SEM. **P<0.01, * P<0.05, WT vs. S2814D. (TIF) [file pone.0058655.s004.tif]
